# Dentigerous Cyst in a Pediatric Patient: A Case Report

**DOI:** 10.7759/cureus.59223

**Published:** 2024-04-28

**Authors:** Aishwarya Kothari, Vinod V Shinde, Mayur Ingale, Sunanda Devi Putta

**Affiliations:** 1 Department of Otorhinolaryngology, Dr. D.Y. Patil Medical College Hospital and Research Centre, Dr. D.Y. Patil University, Pune, IND

**Keywords:** endoscopic sinus surgery (ess), functional endoscopic sinus surgery (fess), caldwell luc approach, 3rd molar, dentigerous cyst (dc)

## Abstract

One of the most prevalent types of odontogenic cysts is a dentigerous cyst, which is usually connected to the crown of an immature tooth. We report the case of an 11-year-old boy, who had a swelling over his left cheek, which was determined to be a dentigerous cyst by radiological imaging and clinical examination. Over the course of two months, the peanut-sized mass grew to 3x2 cm. A massive, well-defined cystic lesion connected to an unerupted premolar tooth was found on a CT scan of the left maxillary alveolar arch and sinus floor. Under general anesthesia, the patient had a Caldwell-Luc surgery to remove the cyst. In order to avoid difficulties related to cyst formation, which can invade surrounding tissues and even result in cancer if left untreated, early detection using radiological imaging is essential. Complete excision of the cyst is the treatment, particularly for big lesions, in order to limit morbidity and lower the likelihood of aggressive behavior. This case emphasizes the necessity of thorough examination and surgical intervention when necessary, underscoring the significance of early identification and adequate therapy to minimize potential problems related to dentigerous cysts. In cases of dentigerous cysts, early intervention, and appropriate surgical procedures are critical to reducing morbidity and improving patient outcomes.

## Introduction

An epithelial-lined pathological cavity is called a cyst. The fibrocollagenous connective tissue that envelops the epithelium can originate from several origins. The basal epithelium of the stomodeum gives rise to the odontogenic epithelium, which in turn gives rise to odontogenic cysts [1]. The primary reason these bones are being destroyed is because of a frequent collection of tumors called odontogenic cysts that develop in the maxilla and mandible [2]. About 20% to 24% of all epithelium-lined cysts of the jaws are dentigerous cysts, the most prevalent kind of developing odontogenic cyst [3].

A dentigerous cyst is described as one that develops from the follicle separating from the surrounding tissue of an unerupted tooth's crown [1]. Comparatively, males are twice as affected as females [3]. While any tooth may be impacted by the cyst, mandibular third molars are the most frequently afflicted [4]. Large dentigerous cysts may be linked to the painless extension of the jaw in the affected area. Dentigerous cysts can develop to a significant size. Face asymmetry is caused by extensive lesions [5].

Here, we present a case of an 11-year-old male who presented with left cheek swelling, which upon investigation was promptly diagnosed as a dentigerous cyst and managed with excision by doing the Caldwell-Luc procedure.

## Case presentation

**Figure 1:**
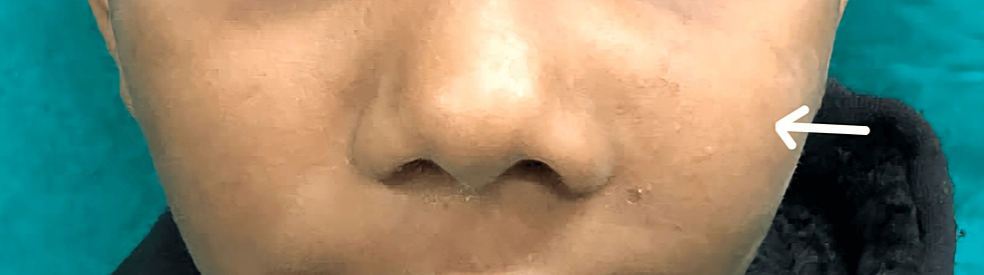
Swelling present over the left cheek (white arrow)

An 11-year-old male patient presented with swelling over the left cheek for two months (Figure 1).

It was sudden in onset and gradually progressive in nature over one month. Initially, the swelling was that of the size of a peanut but later progressed to the present size of approximately 3×2 cm. He did not have any other similar swelling over the face or any other part of the body. There were no complaints of bleeding or discharge from the swelling. There was no history of any long-standing chronic illness. Past and family history were not significant.

**Figure 2:**
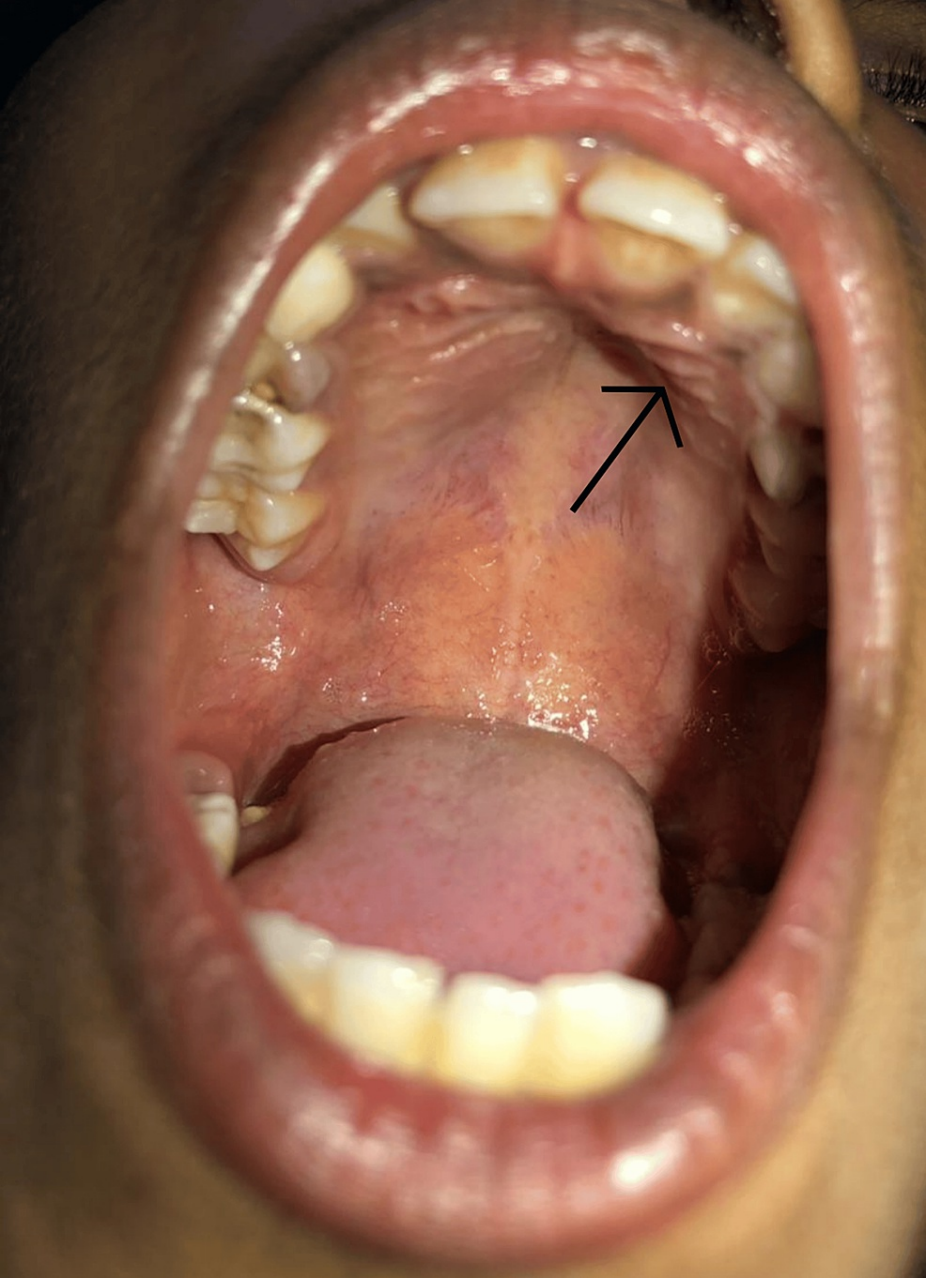
Bulge over the hard palate on the inner aspect of the swelling

External examination of the left cheek revealed a swelling of approximately 3x2 cm that extended medially from the left ala of the nose to 3 cm medial to the left ear's tragus laterally. The enlargement reached the inferior left maxillary gingiva and continued superiorly 0.5 cm below the left infraorbital margin. On the inner aspect of the swelling, as seen in the examination of the oral cavity, there was a bulge over the hard palate (Figure 2).

On palpation, all the inspectory findings were confirmed. There were no other signs of inflammation over the external surface of the swelling. On oral examination, there was a solitary, smooth, hard swelling present over the hard palate on the left side. It was non-tender with no local rise in temperature. The skin over the swelling was normal. The rest of the oral cavity was normal.

**Figure 3:**
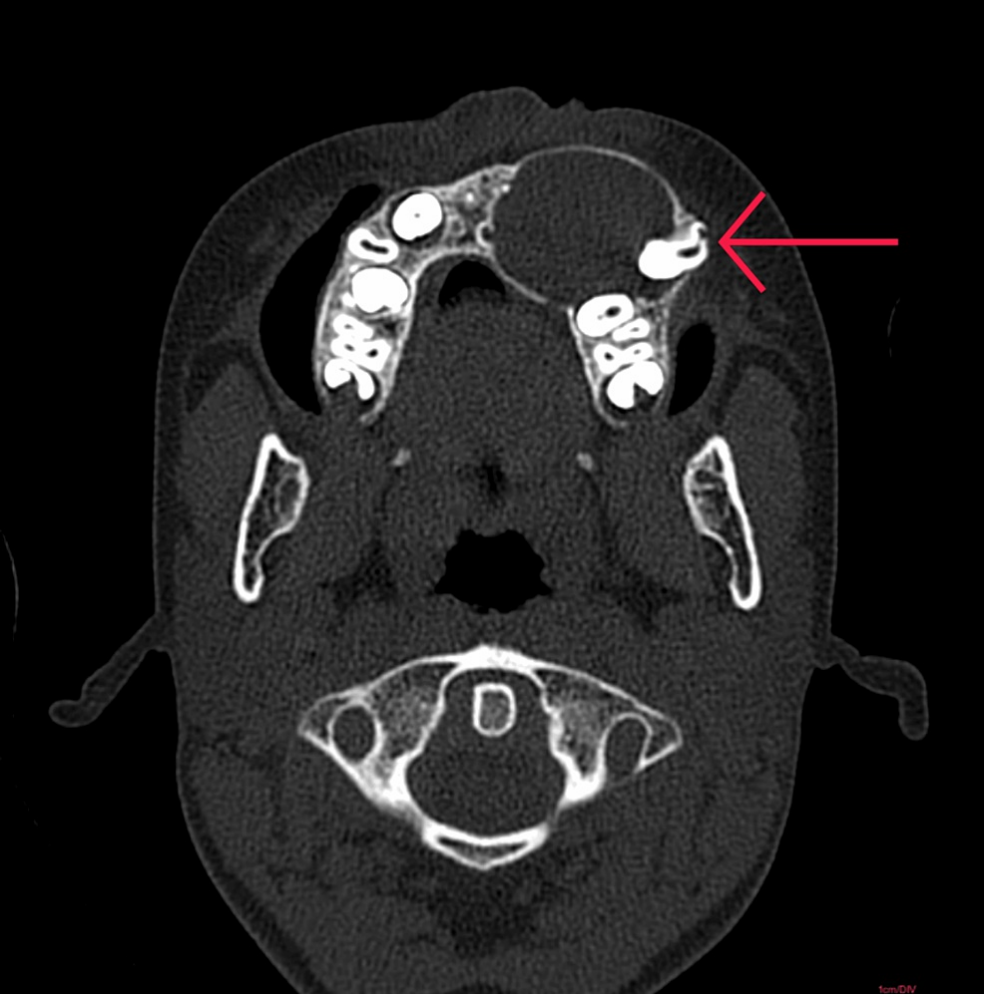
CT axial section of paranasal sinus showing a well-defined expansile cystic lesion with peripheral thin rim enhancement in the left side of the maxillary alveolar arch in relation to the crown of the unerupted premolar tooth

All routine blood investigations were done and were within normal limits. CT scan of the paranasal sinus (Figure 3) revealed a large, well-defined expansile cystic lesion on the left side of the maxillary alveolar arch and anterior part of the floor of the left maxillary sinus in relation to the crown of the unerupted premolar tooth likely to be a dentigerous cyst.

After obtaining fitness for surgery from the anesthesiologist and written informed consent from the patient and relatives, the patient was taken up for the left Caldwell-Luc procedure under general anesthesia. An incision was made on the left upper gingiva-buccal area from the left upper lateral incisor to the left upper first molar. The anterior wall of the maxillary sinus was breached.

The misplaced tooth along with its cystic wall was visualized in the left maxillary sinus and was excised. The rest of the maxillary sinus was inspected and was normal. Suturing was done in layers. Hemostasis was achieved, and the postoperative period was uneventful.

**Figure 4:**
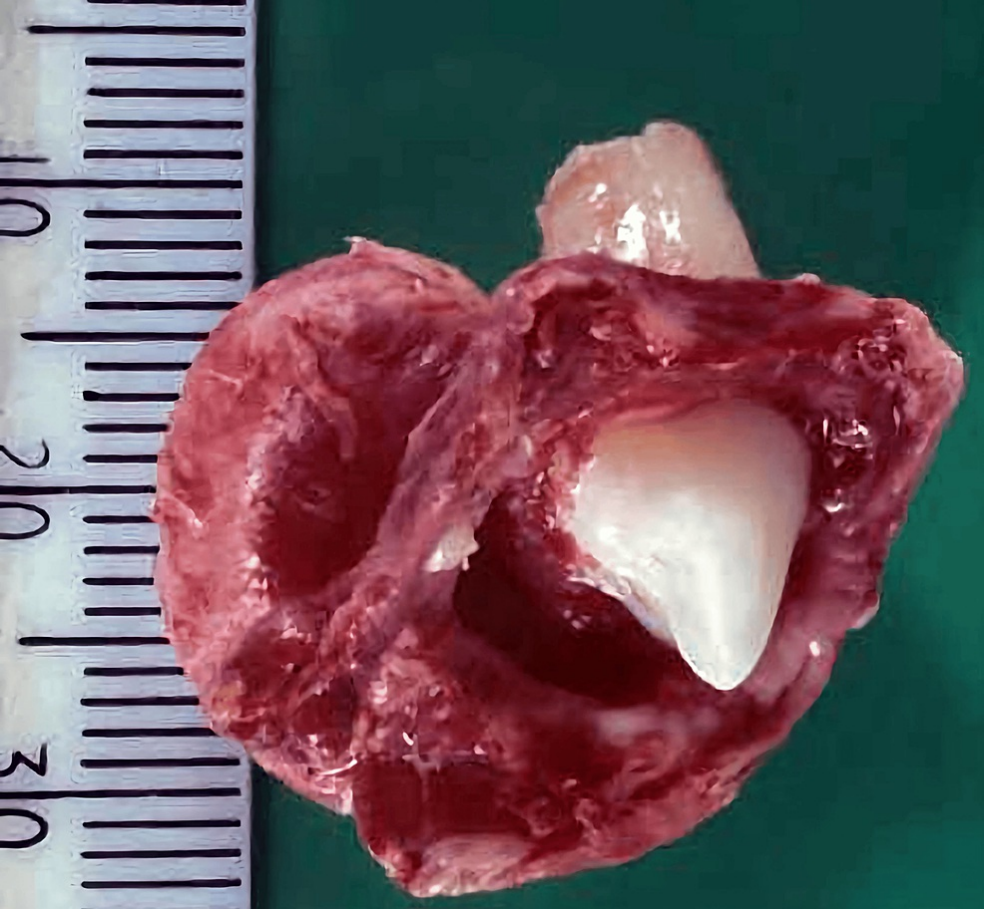
Excised mass showing an unerupted premolar tooth and cystic contents

Further, the excised mass (Figure 4) was sent for histopathological testing.

**Figure 5:**
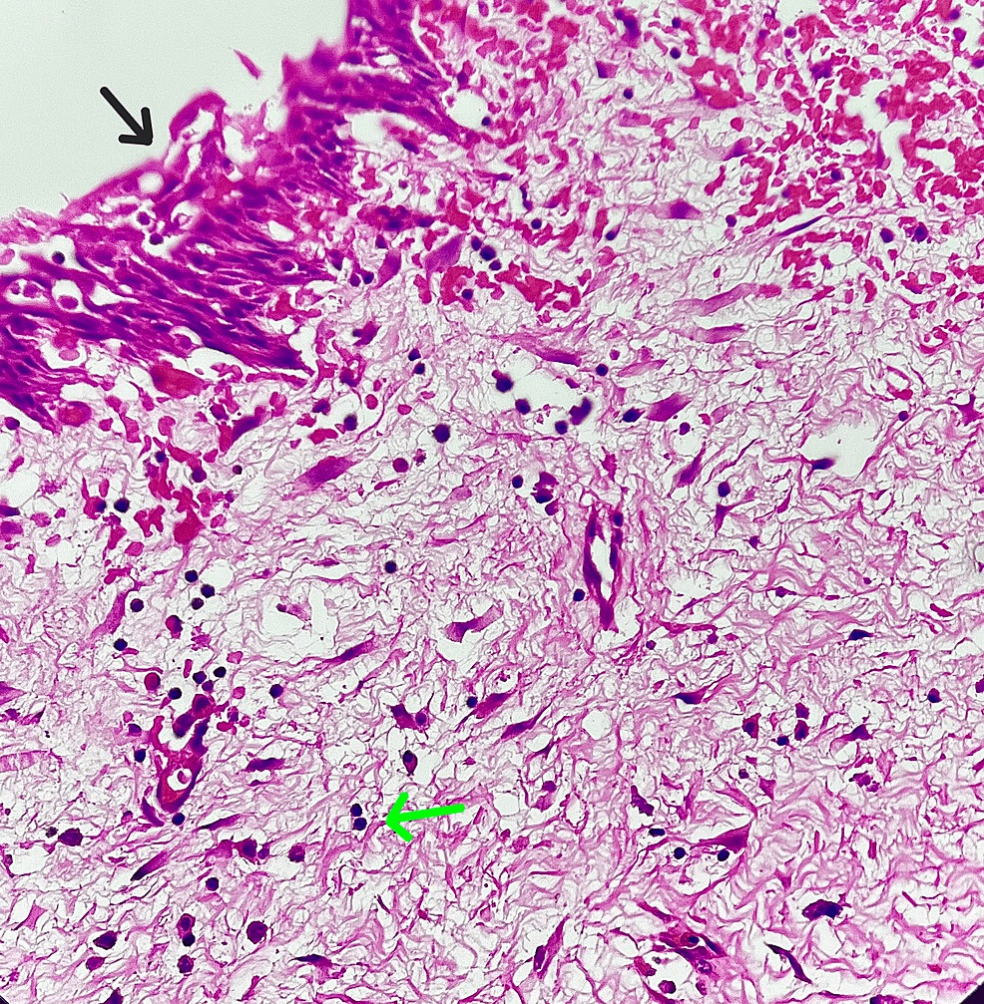
HPE showing non-keratinizing stratified squamous epithelium (black arrow) and fibrous connective tissue with chronic inflammatory cells (green arrow) HPE: histopathology

Specimen for histopathology (HPE) on examination (Figure 5) showed the cyst lined by non-keratinizing stratified squamous epithelium with loss of basal palisading. The subepithelium showed fibrous connective tissue along with secondary changes and a moderate to intense secondary chronic inflammatory cells infiltrate was noted.

## Discussion

The extensive epithelium that proliferates in the bone during tooth development and along the lines where the surfaces of embryologic jaw processes merge may be linked to the incidence of cysts in the jaws [6].

Some of the odontogenic cysts are classified as ‘developmental’ in terms of their pathophysiology while others are classified as ‘inflammatory’ [7].

The maxilla (30%) and mandible (70%) are the two regions where DCs are most commonly seen. People in their 20s and 30s have the highest incidence of DC (23% and 20%, respectively). Very few dentigerous cysts occur in the first 10-12 years of life, as was the situation with our patient [8].

Dentigerous cysts are typically seen during routine radiography exams or when films are obtained to ascertain why a tooth is not erupting. Large lesions occasionally have a scalloping multilocular pattern, although they are invariably radiolucent and typically unilocular [9].

There are several possibilities, however, the precise etiology of this cyst is still unclear. According to the "intrafollicular theory," a dentigerous cyst results from fluid buildup between the epithelium's inner and outer surfaces. This build-up happens as the crown is forming. Enamel hypoplasia theory is the second theory. It indicates that stellate reticulum degradation precedes the cyst's formation. According to Main's theory, the cyst develops as a result of the hydrostatic pressure that an impacted tooth applies to the follicle, causing the impacted crown to separate from the surrounding follicle [2].

Often, dentigerous cysts are found during radiographs ordered to look into dental malalignment, tooth loss, or failed tooth eruption. The cyst often doesn't cause any pain or discomfort unless it develops a secondary infection [4].

It is discovered as an unintentional radiological finding while looking into tooth eruption failure, missing teeth, malalignment, or the emergence of secondary acute inflammation, infection, or swelling. It manifests as a well-defined, unilocular, typically symmetric radiolucency surrounding the crown of a prominent radiopaque impacted tooth, which is typically found on the maxillary sinus floor. Orthopantomogram (OPG), plain skull radiography, and paranasal sinus X-ray (Waters' view) are all quick, low-cost techniques that may be used in standard clinical settings. Cysts can differ greatly in size and extent, and ectopic teeth might be seen near the eye; in these cases, standard radiographs might not be enough to determine the size of the cysts or their distance from other important anatomical structures. Therefore, it is best to use computed tomography (CT) [10].

Differential diagnoses of a dentigerous cyst can be odontogenic keratocyst, ameloblastic fibroma, fibro-odontoma, early stages of Gorlin cyst/calcifying epithelial odontogenic tumor (CEOT), unicystic ameloblastoma, and adenomatoid odontogenic tumor [11].

Histologically, the dentigerous cyst wall is made of thin connective tissue that has a layer of stratified squamous epithelium bordering the cyst lumen and varied numbers of islands of odontogenic epithelium. Infiltration of connective tissue by inflammatory cells is a frequent occurrence. The epithelial lining contains unusual, frequently curved, hyaline structures that are most likely of hematogeneous origin. These entities are known as Rushton bodies. The yellow, watery fluid that makes up the cyst lumen occasionally has a blood tint to it [3].

Treatment for dentigerous cysts is based on the cyst's size, location, and deformity, and typically involves varying the amount of bone removed to guarantee complete eradication of the cyst, particularly in big instances [12]. As suggested in the literature, because of the size of the cyst, instead of the endoscopic approach, the Caldwell-Luc approach was performed [8].

When the tooth and cyst are close to the osteomeatal complex, an entirely transnasal endoscopic procedure may be utilized, ideally via a middle meatal antrostomy. When the tooth is too big to be removed with a middle meatotomy that is the right size or when it is too close to the orbital floor or nasolacrimal duct, a combined endoscopic and Caldwell-Luc technique might be explored [13]. For a dentigerous cyst in the maxillary sinus, the Caldwell-Luc technique is used to enucleate the cyst and extract the related tooth [11].

The dentigerous cyst has the capacity to develop into an aggressive lesion [3].

If it is neglected for more than a year, it may grow and encroach on nearby tissues such as the orbit, hard palate, alveolar arch, and nasal septum [11]. Mucoepidermoid carcinoma, epidermoid carcinoma, or ameloblastoma are possible adverse outcomes [3].

## Conclusions

In summary, this case placed emphasis on identifying the type of cyst after obtaining a thorough clinical history and radiological investigations. Dentigerous cysts are cysts formed by unerupted teeth attached to the cement-enamel junction, mostly seen between the second and third decades of life. A proper understanding of the nature of the cyst can help in early diagnosis and management, thereby significantly reducing morbidity in patients, especially those in the pediatric age group. In this case, we have placed emphasis on the Caldwell-Luc technique as a surgical approach for the excision of a cyst with an unerupted tooth and its cystic contents. To provide individualized treatment for the best possible patient outcomes, a multidisciplinary approach is imperative in negotiating the complexities of a cyst.
